# Climate-based dengue model in Semarang, Indonesia: Predictions and descriptive analysis

**DOI:** 10.1016/j.idm.2021.03.005

**Published:** 2021-03-24

**Authors:** Nuning Nuraini, Ilham Saiful Fauzi, Muhammad Fakhruddin, Ardhasena Sopaheluwakan, Edy Soewono

**Affiliations:** a Department of Mathematics, Faculty of Mathematics and Natural Sciences, Institut Teknologi Bandung, Bandung, 40132, Indonesia; b Center for Mathematical Modeling and Simulation, Institut Teknologi Bandung, Bandung, 40132, Indonesia; c Department of Mathematics, Faculty of Military Mathematics and Natural Sciences, The Republic of Indonesia Defense University, IPSC Area, Sentul, Bogor, 16810, Indonesia; d Center for Research and Development, Indonesian Agency for Meteorology, Climatology and Geophysics, Jakarta, 10720, Indonesia

**Keywords:** Dengue, Climate, Host-vector model, Infection rate, Prediction, Descriptive analysis

## Abstract

**Background:**

Dengue is one of the most rapidly spreading vector-borne diseases, which is considered to be a major health concern in tropical and sub-tropical countries. It is strongly believed that the spread and abundance of vectors are related to climate. Construction of climate-based mathematical model that integrates meteorological factors into disease infection model becomes compelling challenge since the climate is positively associated with both incidence and vector existence.

**Methods:**

A host-vector model is constructed to simulate the dynamic of transmission. The infection rate parameter is replaced with the time-dependent coefficient obtained by optimization to approximate the daily dengue data. Further, the optimized infection rate is denoted as a function of climate variables using the Autoregressive Distributed Lag (ARDL) model.

**Results:**

The infection parameter can be extended when updated daily climates are known, and it can be useful to forecast dengue incidence. This approach provides proper prediction, even when tested in increasing or decreasing prediction windows. In addition, associations between climate and dengue are presented as a reversed slide-shaped curve for dengue-humidity and a reversed U-shaped curves for dengue-temperature and dengue-precipitation. The range of optimal temperature for infection is 24.3–30.5 °C. Humidity and precipitation are positively associated with dengue upper the threshold 70*%* at lag 38 days and below 50 mm at lag 50 days, respectively.

**Conclusion:**

Identification of association between climate and dengue is potentially useful to counter the high risk of dengue and strengthen the public health system and reduce the increase of the dengue burden.

## 1 Introduction

Dengue fever is a disease caused by virus infection, which has become a public health threat where carrier vectors are present. Dengue infectious agent belongs to a *flavivirus*, that is transmitted to human by the bite of mosquito from the genus *Aedes*. Dengue outbreaks occurred in more than 100 countries, and the dengue burden was mostly concentrated in tropics and subtropics regions. More than 2.5 billion people or close to half of the world population are at risk of being exposed by dengue. Approximately 390 million (range 284–528 million) people have been positively infected by dengue annually (Liu-Helmersson et al., 2014; WHO, 2011; Williams et al., 2015). The globalization process, which has been increased global connectivity and human mobility, affected the distribution of virus and its transmission vector. It subsequently triggered the spread of dengue incidence in more extensive geographic areas, resulting in urging global worrisome (Liew et al., 2016; Russel et al., 2009).

Female *Aedes* mosquitoes are predominantly infected with dengue viruses after they bit infectious hosts during the blood meal. It takes 15 days (on average) at the favorable environmental condition for a virus to replicate, mature, and move to the salivary glands until the mosquito can transmit the virus and infect another susceptible human, probably throughout the mosquito’s life span (Ebi & Nealon, 2016; Yang et al., 2011). After the virus is transmitted into a susceptible human by an infectious mosquito, the person undergoes an incubation period, which varies within 4–10 days. Asymptomatic with mild symptoms occurs in most cases. Still, on the other hand, the disease can involve the dengue hemorrhagic fever (DHF) or dengue shock syndrome (DSS) with case-fatality rates close to 1 percent (Andraud et al., 2013; Putra & Nuraini, 2017). The infectious period varies between 3 and 7 days as the manifestation of dengue symptoms (Yang et al., 2011). Infected symptomatic and asymptomatic hosts are carriers of the virus for susceptible vectors. After that, the recovered human develops a permanent immunity against the infecting serotype but only temporary cross-protective immunity to different serotypes (Esteva & Vargas, 1998; Fakhruddin et al., 2019).

Meteorological variables have generally been known as an important parameter in driving the dengue outbreaks. Climate and weather are substantial variables in determining *Aedes* mosquito’s ecology, development, survival, and behavior as the primary vector of dengue (Alkhaldy, 2017). Seasonal variations of environmental climate are also associated with the existence and the expansion of mosquito breeding sites, which support the abundance of vector population on a certain period (Arcari et al., 2007; Ong & Tan, 2019; Williams et al., 2015). Temperature, humidity, and rainfall can directly influence the growth at all phases of mosquito’s life cycle that may indirectly affect the dengue transmission cycle. These variables can not be considered independently but rather as a group that have a cumulative effect upon dengue transmission (Alkhaldy, 2017). Furthermore, the climatic condition also greatly influences the probability of successful virus transmission between host and vector. The entomological parameters regarding mosquito development and virus transmission are climatically sensitive for many reasons (Putra & Nuraini, 2017; Russel et al., 2009). For example, numerous previous researches have identified that variation on temperature affects mosquito vectorial capacity, i.e., frequency of biting activity, vector mortality rate, and virus transmission effectiveness (Li et al., 2017; Liu-Helmersson et al., 2014; Ong & Tan, 2019). Higher temperature shortens the vector development period, increases the intensity of blood-feeding, and decreases the extrinsic incubation period’s duration among female mosquitoes (Ebi & Nealon, 2016; Sharmin et al., 2015). The relative humidity is likewise seen to influence vector competence and biting behavior sensitively, the survival rate of adult mosquito (Ebi & Nealon, 2016; Islam et al., 2018), egg-laying, and mating pattern (Wu et al., 2007). Longer lifespan and further dispersion of dengue vectors under high relative humidity increase the chance of mosquito transmitting dengue virus to more people (Promprou et al., 2005). Besides, the abundance of vector population is affected by the breeding site that the presence is strongly correlated by the rain (Gotz et al., 2017; Li et al., 2017). The more it rains, the more egg-laying zones are available for the mosquito, and thus the size of the vector population increases (Promprou et al., 2005). Nevertheless, temperature conditions above 30 °C can shorten adult mosquito lifespan as does either in very high and very low precipitation that can remove the breeding place of mosquito (Putra & Nuraini, 2017; Sharmin et al., 2015).

Semarang is a lowland area located in the northern coast of Java and one of Indonesia’s riskiest cities with a long record of dengue cases annually. Semarang’s tropical climate supports a favorable environment for vector growth and breeding, making dengue fever continue to be a health threat for the public. Some researches have been conducted to explain the dengue occurrences in Semarang. Gotz et al. (Gotz et al., 2017) modified the classical host-vector model of the type SIR-SI with a reduction in mosquito dynamic at its equilibrium. Further, the implementation of an optimal control problem was done to obtain the time-dependent infection rate parameter, which produced proper data fitting and a model for the infection rate parameter as a monthly precipitation function. Putra and Nuraini (Putra & Nuraini, 2017) constructed an early warning model of dengue outbreaks based on the dynamic of the vector population and the host-vector threshold values considering the condition of precipitation and temperature. Furthermore, Fauzi et al. (Fauzi et al., 2019) compared the pattern of dengue transmission in lowland area and highland area in which Semarang was chosen as the representative of lowland area, and also identified seasonal variations of dengue incidence via a continuous sinusoidal function of infection rate and examined the correlation between humidity-incidence and humidity-infection rate.

This paper deals with the development host-vector model for climate-based dengue transmission. Here, mosquito entomological parameters in the biological model were formulated with the assumption that the infection rate parameter from vector to host was time-dependent. The parameter value was obtained from the optimization to minimize the deviation between the output of model and dengue incidence data. This approach was expected to provide novelty and usefulness in the use of mathematical models in predicting dengue incidence. The modification was focused on unobservable parameters; one of them was the infection rate, which served as a function of the climate variables. Subsequently, it could be used to predict the dengue incidence when the updated daily climate conditions were known. Furthermore, the associations between climatic factors and dengue incidences were investigated to describe the conditions when the dengue cases mostly occurred. The model was able to be used as a predictor of the dengue epidemic in the future and as an early warning system for the local health agency to improve dengue prevention strategies and vector control.

## 2 Material and methods

### 2.1 Collected data

**Fig. 1:**
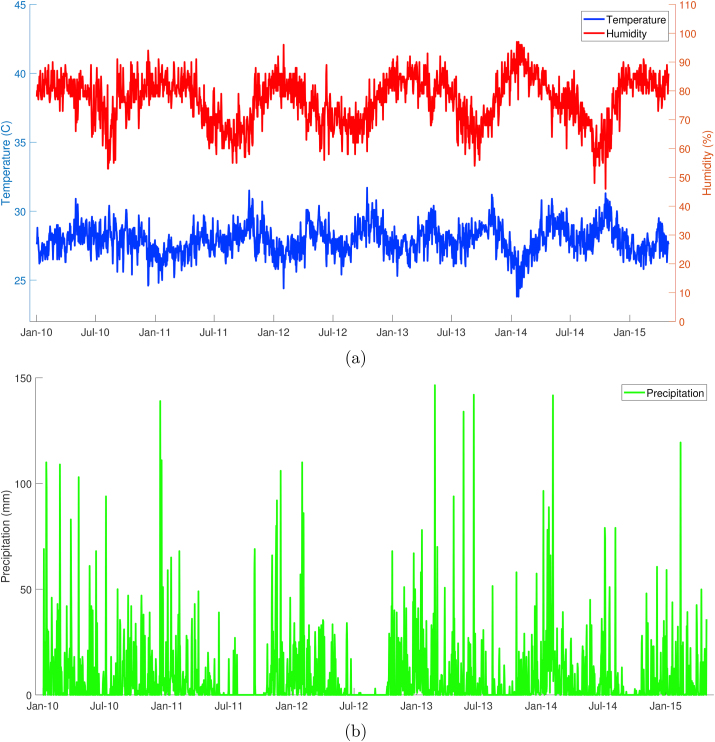
Climate conditions in Semarang: (a) temperature and humidity; (b) precipitation.

Semarang is the capital and largest city in Central Java province that covers a 373.67 square kilometers area. The downtown is located 2.45 m above sea level, with the total residents of about 1.5 million. Semarang features a tropical climate zone where *Aedes* mosquito can be found, and therefore this condition supports the transmission of dengue virus. Meteorological data were obtained from the Indonesian Agency for Meteorology, Climatology, and Geophysics (BMKG) and presented in Fig. 1. According to the collected data with the period of January 2010 to April 2015, the rainy season mostly occurred from October to May and had annual cumulative precipitation approximately 2827 mm. The highest precipitation usually occurred in January, with level reaching 754 mm in a month. The lowest rainfall occurred during the dry season (June to September), with precipitation sometimes zero between July and September. The daily average temperature range was 23.8–31.7 °C, and the percentage of daily humidity expands between 46 percent until 97 percent (Pemerintah kota Semarang, 2019).

**Fig. 2:**
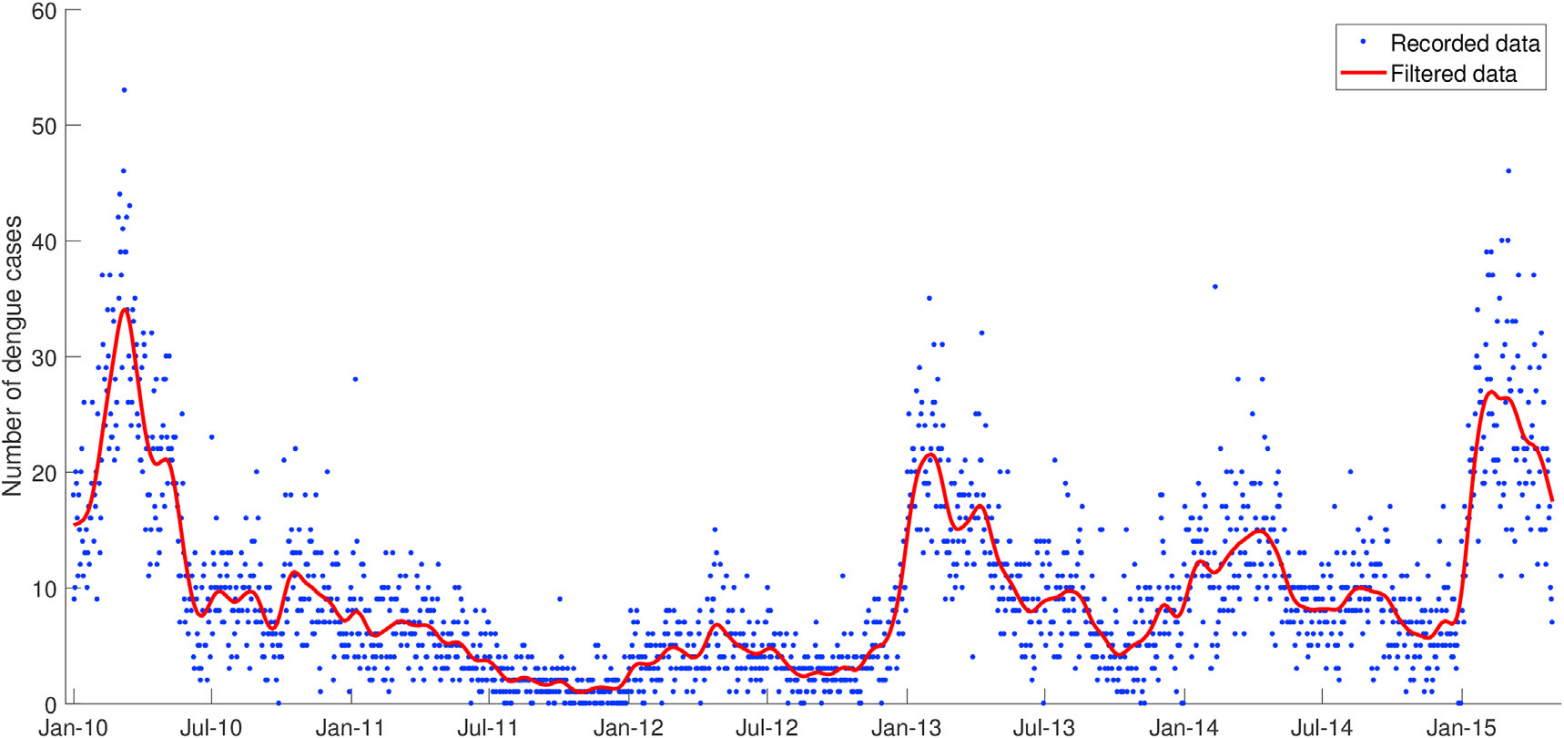
Total number of hospitalized dengue cases in Semarang.

Semarang is known as one of the dengue-endemic areas where high-frequency cases are reported each year. This study is supported by daily dengue data from Semarang Health Office (Dinas Kesehatan). As shown in Fig. 2, the total number of hospitalized dengue incidence has been recorded and is available for the period from January 2010 to April 2015. Collected data is the accumulation of Dengue Fever (DF), Dengue Hemorrhagic Fever (DHF), and Dengue Shock Syndrome (DSS). According to the collected dengue incidence data, the dengue outbreak usually occurred at the beginning of a year from January to March. They could reach 52 suspects of dengue fever in a day. Gaussian filter was implemented to eliminate high-frequency fluctuations in the actual raw dengue data without changing the original data pattern.

### 2.2 Mathematical model

The basic model for the dengue transmission is constructed here with SIR-SI dynamical system in which the human population is divided into three compartments, i.e., Susceptible ${\bar{S}}_{h}$, Infected ${\bar{I}}_{h}$, and Recovered ${\bar{R}}_{h}$, and the vector population is divided into two compartments, i.e., Susceptible ${\bar{S}}_{v}$ and Infected ${\bar{I}}_{v}$ (Esteva & Vargas, 1998). The system is represented by the following equations.

(1) $$\begin{array}{cc} & \frac{d{\bar{S}}_{h}}{dt}=\mu_{h}\left({\bar{N}}_{h}-{\bar{S}}_{h}\right)-\frac{bP_{h}{\bar{S}}_{h}{\bar{I}}_{v}}{{\bar{N}}_{v}}\\ & \frac{d{\bar{I}}_{h}}{dt}=\frac{bP_{h}{\bar{S}}_{h}{\bar{I}}_{v}}{{\bar{N}}_{v}}-\left(\gamma +\mu_{h}\right){\bar{I}}_{h}\\ & \frac{d{\bar{R}}_{h}}{dt}=\gamma {\bar{I}}_{h}-\mu_{h}{\bar{R}}_{h}\\ & \frac{d{\bar{S}}_{v}}{dt}=\mu_{v}\left({\bar{N}}_{v}-{\bar{S}}_{v}\right)-\frac{bP_{v}{\bar{S}}_{v}{\bar{I}}_{h}}{{\bar{N}}_{h}}\\ & \frac{d{\bar{I}}_{v}}{dt}=\frac{bP_{v}{\bar{S}}_{v}{\bar{I}}_{h}}{{\bar{N}}_{h}}-\mu_{v}{\bar{I}}_{v} \end{array}$$

where *μ*_*h*_ and *μ*_*v*_ correspond to the mortality rate for vector and host, respectively. The host population ${\bar{N}}_{h}={\bar{S}}_{h}+{\bar{I}}_{h}+{\bar{R}}_{h}$ and the vector population ${\bar{N}}_{v}={\bar{S}}_{v}+{\bar{I}}_{v}$ are considered constant. The parameters and their biological interpretations are given in Table 1.

**Table 1:** Description of variables and parameters used in mathematical model.

Symbol	Description	Unit
${\bar{N}}_{h}$	The total number of host population	human
${\bar{N}}_{v}$	The total number of vector population	mosquito
${\bar{S}}_{h}$	The number of susceptible host	human
${\bar{I}}_{h}$	The number of infected host	human
${\bar{R}}_{h}$	The number of recovered host	human
${\bar{S}}_{v}$	The number of susceptible vector	mosquito
${\bar{I}}_{v}$	The number of infected vector	mosquito
*μ*_*h*_	Host natural birth and mortality rate	1/day
*μ*_*v*_	Vector mortality rate	1/day
*b*	Biting rate	1/day
*P*_*h*_	Probability of transmission from vector to host	–
*P*_*v*_	Probability of transmission from host to vector	–
*γ*	Host recovery rate	1/day

The differential equation system is normalized by dividing every state variable with the total number of the human population (${\bar{N}}_{h}$) or the total number of mosquito population (${\bar{N}}_{v}$) depending on each variable’s classification.

$$S_{h}=\frac{{\bar{S}}_{h}}{{\bar{N}}_{h}},\;I_{h}=\frac{{\bar{I}}_{h}}{{\bar{N}}_{h}},\;R_{h}=\frac{{\bar{R}}_{h}}{{\bar{N}}_{h}},\;S_{v}=\frac{{\bar{S}}_{v}}{{\bar{N}}_{v}},\;I_{v}=\frac{{\bar{I}}_{v}}{{\bar{N}}_{v}}$$

Each normalized variable denotes the proportion of the total population of human or population of mosquito. The five-dimensional dynamical system *x*′ = *f* (*t*, *x*) for the state variable $x=\left(S_{h},I_{h},R_{h},S_{v},I_{v}\right)$ is reduced to be a three-dimensional dynamical system using the fact that the total population of human and mosquito are constant, i.e., for *S*_*h*_ = 1 − (*I*_*h*_ + *R*_*h*_) and *S*_*v*_ = 1 − *I*_*v*_. The dynamical system obtained from normalization and reduction is shown in the following equations:

(2) $$\begin{array}{cc} & \frac{dI_{h}}{dt}=bP_{h}\left(1-I_{h}-R_{h}\right)I_{v}-\left(\gamma +\mu_{h}\right)I_{h}\\ & \frac{dR_{h}}{dt}=\gamma I_{h}-\mu_{h}R_{h}\\ & \frac{dI_{v}}{dt}=bP_{v}\left(1-I_{v}\right)I_{h}-\mu_{v}I_{v} \end{array}$$

The dynamical system has a disease free equilibrium (DFE), $\left(I_{h}^{\circ },R_{h}^{\circ },I_{v}^{\circ }\right)=\left(0,0,0\right)$, and a endemic equilibrium (EE),

$$\left(I_{h}^{*},R_{h}^{*},I_{v}^{*}\right)=\left(\frac{\mu_{h}\delta }{bP_{v}\zeta },\frac{\gamma \delta }{bP_{v}\zeta },\frac{\mu_{h}\delta }{bP_{h}\eta }\right),$$

where $\zeta =\left(bP_{h}+\mu_{h}\right)\left(\gamma +\mu_{h}\right)$, $\eta =bP_{v}\mu_{h}+\mu_{v}\left(\gamma +\mu_{h}\right)$ and $\delta =b^{2}P_{h}P_{v}-\mu_{v}\left(\gamma +\mu_{h}\right)$

A basic reproduction number, denoted by *R*_◦_, can be obtained using Next Generation Matrix (NGM). The infected subsystem $x^{T}=\left(I_{h},I_{v}\right)$ is considered and linearized at DFE to obtain a Jacobian matrix. The dominant eigenvalue is the basic reproduction number which is shown as:

$$R_{\circ }=\frac{b^{2}P_{h}P_{v}}{\mu_{v}\left(\gamma +\mu_{h}\right)}$$

The disease free equilibrium (DFE) is always exist. However, by considering $I_{h}^{*}$ and by using the fact that *μ*_*h*_, *b*, *P*_*v*_ and *ζ* are positive, we require the condition *δ* > 0 or equivalently *R*_◦_ > 1 for the existence of endemic equilibrium (EE). The stability analysis at both DFE and EE are presented in Theorem 2.1 and Theorem 2.2, respectively.

Theorem 2.1 *If* *R*_◦_ < 1, *then disease free equilibrium is locally asymptotically stable*.

Proof. The Jacobian matrix of the differential system at the DFE is given by:

$$J^{\circ }=\left[\begin{array}{ccc} -\left(\gamma +\mu_{h}\right) & 0 & bP_{h}\\ \gamma & -\mu_{h} & 0\\ bP_{v} & 0 & -\mu_{v} \end{array}\right]$$

The characteristic polynomial of matrix *J*^◦^ is *P*(*λ*) = *λ*^3^ + *a*_1_*λ*^2^ + *a*_2_*λ* + *a*_3_ where:

$$\begin{array}{cc} & a_{1}=2\mu_{h}+\gamma +\mu_{v}\\ & a_{2}=\mu_{h}\left(\mu_{h}+\gamma +\mu_{v}\right)-\mu_{v}\left(\gamma +\mu_{h}\right)\left(R_{\circ }-1\right)\\ & a_{3}=-\mu_{h}\mu_{v}\left(\gamma +\mu_{h}\right)\left(R_{\circ }-1\right) \end{array}$$

If all parameters are positive and *R*_◦_ < 1, then *a*_3_ > 0 and *a*_1_*a*_2_ − *a*_3_ > 0. According to *Routh-Hurwitz*’s stability criterion, all roots of the polynomial have negative real part. Thus, the disease free equilibrium is locally asymptotically stable.

Theorem 2.2 *If* *R*_◦_ > 1, *then endemic equilibrium is locally asymptotically stable*.

*Proof*. The Jacobian matrix of the differential system at the EE is given by:

$$J^{*}=\left[\begin{array}{ccc} \frac{\mu_{h}\delta }{\eta }-\left(\gamma +\mu_{h}\right) & \frac{\mu_{h}\delta }{\eta } & bP_{h}\left(1+\delta \left(\frac{\mu_{h}+\gamma }{bP_{v}\zeta }\right)\right)\\ \gamma & -\mu_{h} & 0\\ bP_{v}\left(1+\frac{\mu_{h}\delta }{bP_{h}\eta }\right) & 0 & \frac{\mu_{h}\delta }{\zeta }-\mu_{v} \end{array}\right]$$

The characteristic polynomial of matrix *J*∗ is *Q*(*λ*) = *b*_1_*λ*^3^ + *b*_2_*λ*^2^ + *b*_3_*λ* + *b*_4_ where:

$$\begin{array}{cc} & b_{1}=\eta \zeta \\ & b_{2}=\mu_{h}\mu_{v}\nu \left(\eta +\zeta \right)\left(R_{\circ }-1\right)+\eta \zeta \left(\gamma +2\mu_{h}+\mu_{v}\right)\\ & b_{3}=\mu_{h}\mu_{v}\nu \left[\nu \left(bP_{v}\mu_{h}+\zeta +\gamma \mu_{v}\right)+b^{2}P_{h}P_{v}\mu_{h}+\eta \mu_{h}+\zeta \mu_{v}\right]\left(R_{\circ }-1\right)+\eta \zeta \mu_{h}\left(\nu +\mu_{v}\right)\\ & b_{4}=\mu_{h}\mu_{v}\nu \left(\nu \eta \right)\left(bP_{h}+\mu_{h}\right)\left(R_{\circ }-1\right) \end{array}$$

with *ν* = *γ* + *μ*_*h*_. The premise *R*_◦_ > 1 implies *b*_1_, *b*_2_, *b*_3_, *b*_4_ > 0 and *b*_2_*b*_3_ − *b*_1_*b*_4_ > 0. According to *Routh-Hurwitz*’s stability criterion, all roots of the polynomial have negative real part. Thus, the endemic equilibrium is locally asymptotically stable.

### 2.3 Integration

The model will be improved by accommodating the meteorological parameters, i.e., temperature, humidity, and precipitation. The relation between model and climate variables is connected in transmission and vector entomological parameters. Parameters *b*, *P*_*v*_, and *μ*_*v*_ were assumed to be influenced by temperature conditions where the function of temperature parameters was obtained from Yang et al. (Yang et al., 2011). Parameters *μ*_*h*_ and *γ* were constant, where the values were obtained from the literature (Fauzi et al., 2019; Gotz et al., 2017). Parameter *bP*_*h*_ = *β*_*h*_, called infection rate, is time-dependent, which is obtained from optimization to minimize the error between the result of model simulation and dengue incidence data.

The host population is considered constant, and its value approximates the total population in Semarang at 1.5 million inhabitants. We used the average Indonesian lifespan for the value of host expected lifespan at 65 years. The vector population’s parameters and the virus transmission (vector mortality rate, vector biting frequency, and the probability of successful transmission from host to vector) were associated with temperature. To estimate time-dependent infection rate parameter, we fit the biological model to the smoothed daily incidence data recorded in Semarang from January 2010 to April 2015 using Spiral Dynamic Optimization with cost functional minimizing the deviation between dengue data and numerical result (Tamura & Yasuda, 2011). The initial number of infected humans was obtained from the daily dengue data taking the number of incidences on the first day of January 2010 with *I*_*h*_(0) = 10^−5^ cases per total population. We assumed that initially, 56.9*%* of the human population in Semarang were susceptible to dengue virus infection, and the ratio of infected mosquitoes was three times the ratio of the infected human. The summary of parameter’s value used in simulation was presented in Table 2.

**Table 2:** The parameter’s value used in the numerical simulation.

Symbol	Value	References
*μ*_*h*_	$1/\left(65\cdot 315\right)$	Fauziet al., 2019, Gotzet al., 2017
*μ*_*v*_	0.8692–0.1590 ⋅ *T* + 0.01116 ⋅ *T*^2^ − 3.408 ⋅ 10^−4^ ⋅ *T*^3^ + 3.809 ⋅ 10^−6^ ⋅ *T*^4^	Liu-Helmerssonet al., 2014, Putra and Nuraini, 2017, Yanget al., 2011
*b*	0.0043 ⋅ *T* + 0.0943	Liu-Helmerssonet al., 2014, Putra and Nuraini, 2017, Yanget al., 2011
*P*_*v*_	$0.0729\cdot T-0.9037\left(12.4^{\circ }\leq T\leq 26.1^{\circ }\right)$ or $1\left(26.1^{\circ }<T\leq 32.5^{\circ }\right)$	Liu-Helmerssonet al., 2014, Putra and Nuraini, 2017, Yanget al., 2011
*β*_*h*_	estimated	–
*γ*	1/30	Gotz et al. (2017)
*S*_*h*_(0)	56.9*%*	Andraudet al., 2013, Fauziet al., 2019
*I*_*v*_(0)	3 ⋅ *I*_*h*_(0)	–
*N*_*s*_	1.5 ⋅ 10^6^	–

The cross-correlation was implemented to determine the time lag from the closest relationship between infection rate and each climate factor. We used data with the time window from May 1, 2010, until April 31, 2015. Moreover, the autocorrelation function amounts to determining the time lag when the current data is strongly affected by the previous data. Here we designed an Autoregressive Distributed Lag (ARDL) model with a time lag of infection rate *β*_*h*_ resulted in optimization as a function of temperature (*T*), humidity (*H*), and precipitation (*U*). The model is *autoregressive*, in the sense that the lagged value of itself influence *β*_*h*_. It also has *distributed lag* component, in the form of successive lags of the predictor variables, i.e., *T*, *H*, and *U*. The equation is derived from a direct association between infection rate and climate factors.

$$\beta_{h}\left(t\right)=a_{0}+\overset{k_{1}}{\underset{i=1}{\sum }}b_{i}\cdot \beta_{h}\left(t-i\right)+\overset{k_{2}}{\underset{i=0}{\sum }}c_{i}\cdot T\left(t-i\right)+\overset{k_{3}}{\underset{i=0}{\sum }}d_{i}\cdot H\left(t-i\right)+\overset{k_{4}}{\underset{i=0}{\sum }}e_{i}\cdot U\left(t-i\right)+\varepsilon$$

The error vector obtained in the fitting process is denoted by *ε*. The parameters *a*_0_, *b*_*i*_, *c*_*i*_, *d*_*i*_ and *e*_*i*_ indicate the baseline, the coefficient for previous infection dependence, the coefficient for temperature dependence, humidity dependence, and precipitation dependence, respectively. The parameters *k*_2_, *k*_3_, and *k*_4_ are time lags obtained from cross-correlation, and *k*_1_ is found from partial autocorrelation. The last three parameters also represent the *marginal effect* of temperature, humidity, and precipitation to the infection. Furthermore, the standard least square method is implemented to obtain the parameter values.

## 3 Results and Discussion

**Fig. 3:**
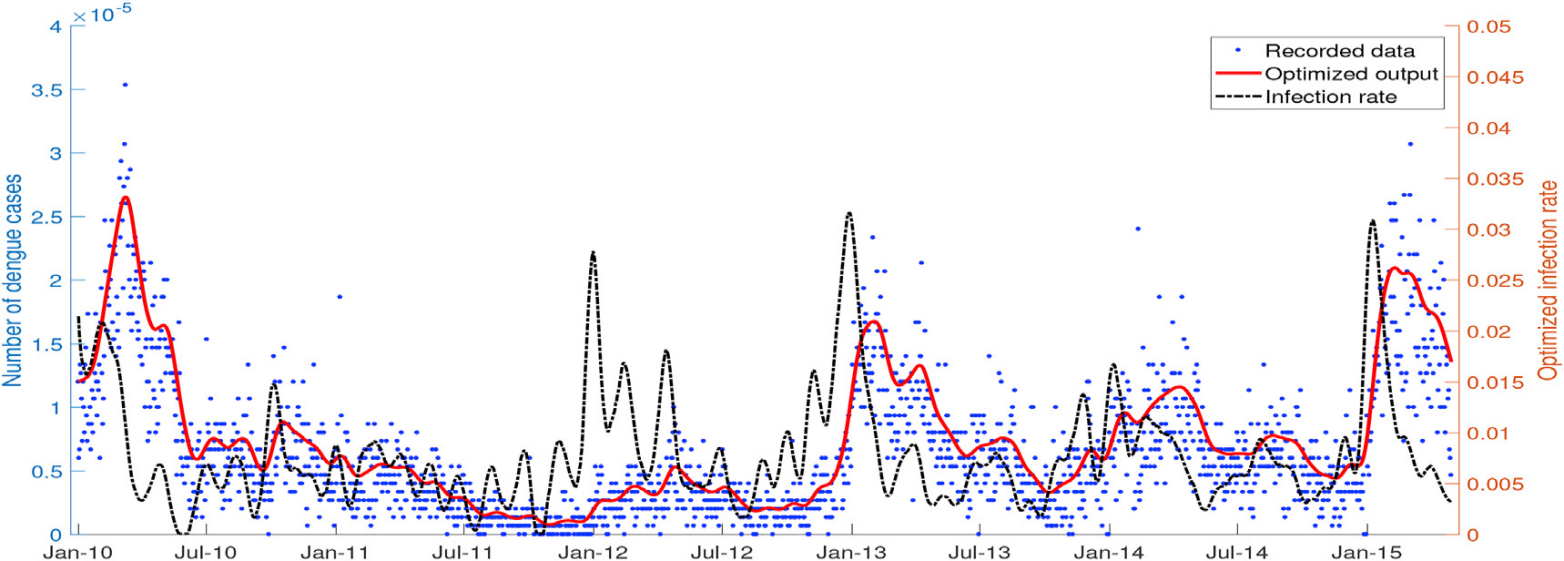
Simulation output of biological model with optimized infection rate *β*_*h*_ as an input.

To obtain the best result of fitting between the output of the biological model and filtered dengue data, the infection rate *β*_*h*_ was treated as a time-independent parameter. For each time interval $\left[t_{n},t_{n+1}\right]$, the value of *β*_*h*_ was obtained from Spiral Dynamic Optimization, which rotated generated random points to its center and eventually minimized the cost functional, i.e., the error between data and simulation. Fig. 3 shows that the actual dengue data is well-approximated by the output of the dengue transmission model, which used an optimized infection rate as an input of numerical simulation.

As a consequence of minimizing deviation and assuming that the infection rate parameter always changes every time, the biological model’s output agreed with dengue data in Semarang and numerical error did not appear significantly in simulation. The value of optimized infection rate *β*_*h*_ exhibited fluctuations but generally had a high value from November to March with the peak in January.

**Fig. 4:**
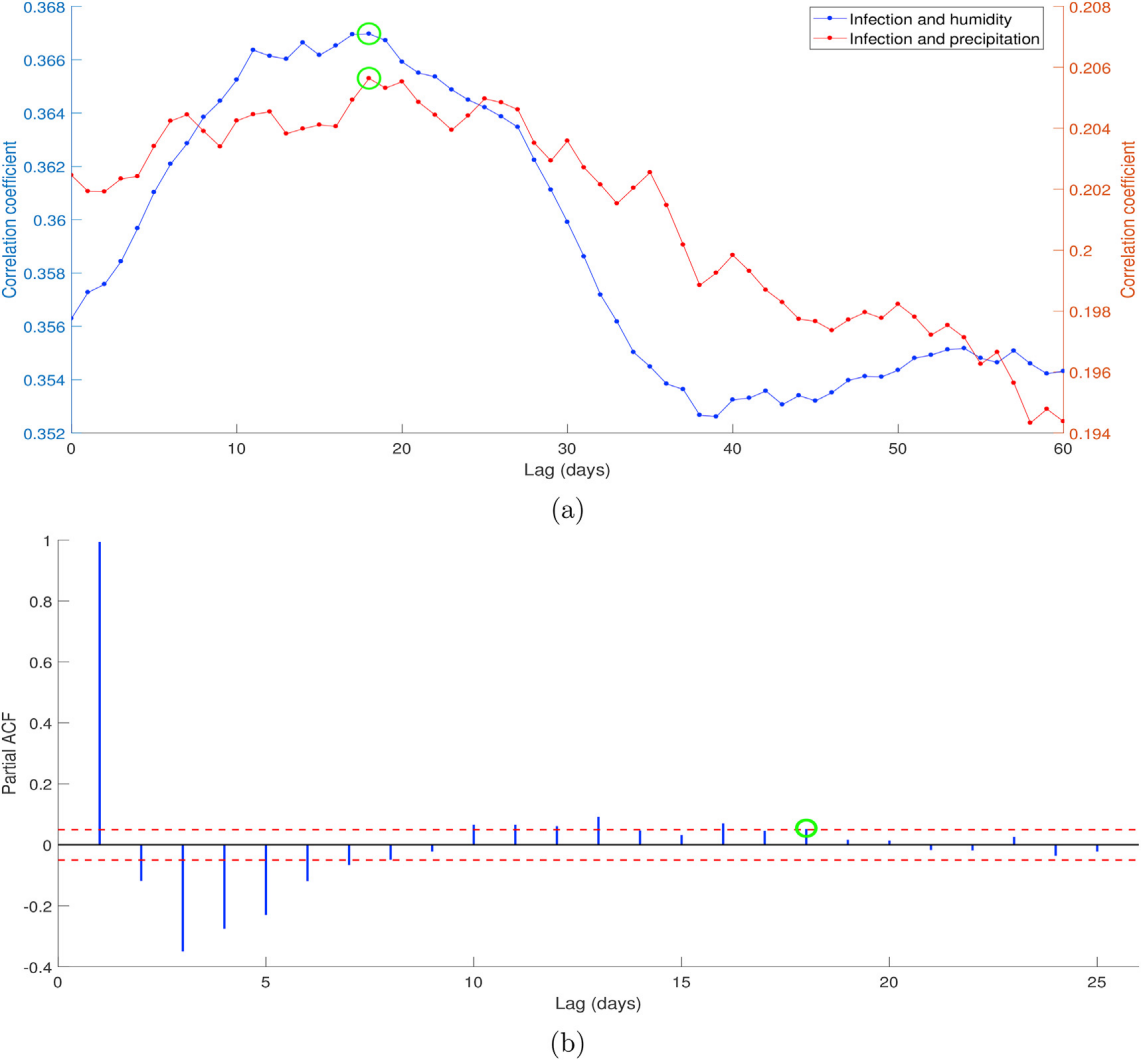
Cross correlation between infection rate *β*_*h*_ and meteorological variables: humidity and precipitation (a), and partial auto correlation of infection rate parameter (b).

According to the cross-correlation result presented in Fig. 4, both infection rate-humidity and infection rate-precipitation showed the time lag of 18 days marked by the green circle. Unlike humidity and precipitation, the Spearman correlation value between infection rate and temperature was not significant $\left(\left|\rho \right|<0.08\right)$, then we ignored the term of temperature on ARDL model (*c*_*i*_ = 0 for all *i*). Although there is a possible association between infection rate and temperature biologically, ignoring temperature does not significantly influence the model based on the statistic perspective because of its low correlation. The time lags between climate variables and dengue cases can be understood when recalling that the environment, including weather conditions, influences dengue vector existence and development. Finally, the partial autocorrelation test on infection rate parameter shows that there are notable spikes which lie outside bands until lag 18 and much lower spikes for the subsequent lags.

Since the time lags of infection rate-humidity and infection rate-precipitation were 18 days, we divided the interval of observation time into *initiating window* [day 1, day 18], *training window* [day 19, day 1825], and *prediction window* [day 1826, day 1945], where the range of prediction time was 120 days (close to 4 months). Furthermore, the *prediction window* was separated into two observation times, *increasing period* (60 days) when the dengue cases were growing and *decreasing period* (60 days) when the dengue cases were shrinking. In the numerical simulation, data of infection rate and climate factors were normalized by subtracting with the mean and dividing with the standard deviation. The parameter values of the ARDL model resulted from the implementation of the ordinary least square method are presented in Table 3.

**Table 3:** Parameters value of ARDL model obtained from least square method.

Index	Parameter *a*	Parameter *b*	Parameter *d*	Parameter *e*
0	−0.00052294	–	0.00319727	−0.00265627
1	–	0.65171741	0.00000715	0.00093975
2	–	0.53265423	−0.00030062	−0.00046613
3	–	0.16792327	0.00399376	0.00301602
4	–	0.03438798	0.00275943	0.00047711
5	–	−0.11585929	0.00344218	0.00273902
6	–	−0.05917912	−0.00525431	−0.00212843
7	–	−0.11625592	0.00304796	−0.00191348
8	–	−0.10037522	−0.00426935	0.00048030
9	–	−0.09324618	0.00298708	−0.00088273
10	–	−0.00638086	−0.00309367	0.00369078
11	–	−0.03559310	0.00175723	−0.00324581
12	–	−0.07290953	−0.00371671	0.00035899
13	–	0.07590263	0.00371369	0.00028356
14	–	0.12553525	−0.00286295	0.00050869
15	–	0.01997659	0.00219171	−0.00030315
16	–	0.01361322	0.00136257	0.00010011
17	–	−0.00777663	−0.00264828	0.00124748
18	–	−0.02271438	−0.00387731	−0.00217288

**Fig. 5:**
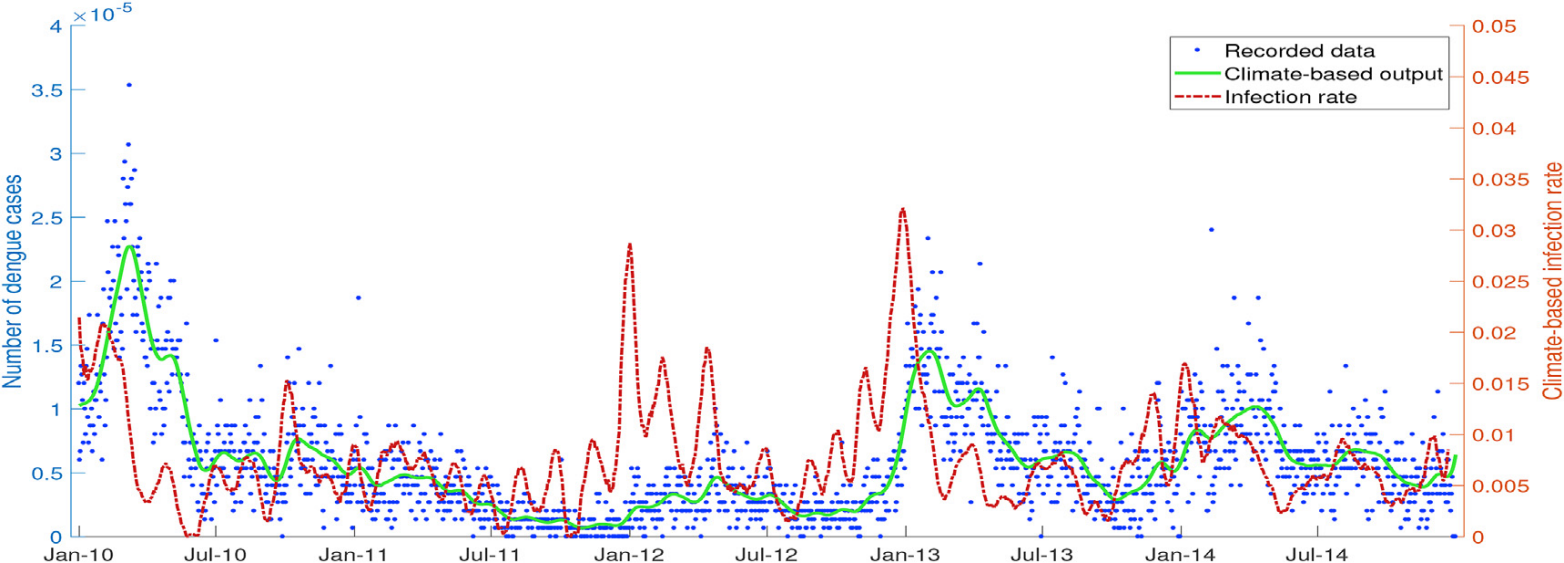
Simulation output of biological model with climate-based infection rate.

In Fig. 5, the fitting result shows small errors between optimized infection rates and climate-based infection rates. The model is also able to catch the peaks of infection rate and long-term seasonality during the *training window*. Although errors appeared at the end of the time interval between output and data, the simulation using a climate-based infection rate still followed the dengue data pattern. The error in the numerical approximation is introduced in one single time step and will be accumulated during the time-stepping procedure, which determines the global error observed after many iteration steps. At time *t*_*n*_, we computed the changes of *I* for time step *t*_*n*_ to *t*_*n*+1_ using the certain value of climate-based infection rate. Since there was a deviation between optimized and climate-based infection rates, each step introduced an error and ended up on a different trajectory.

**Fig. 6:**
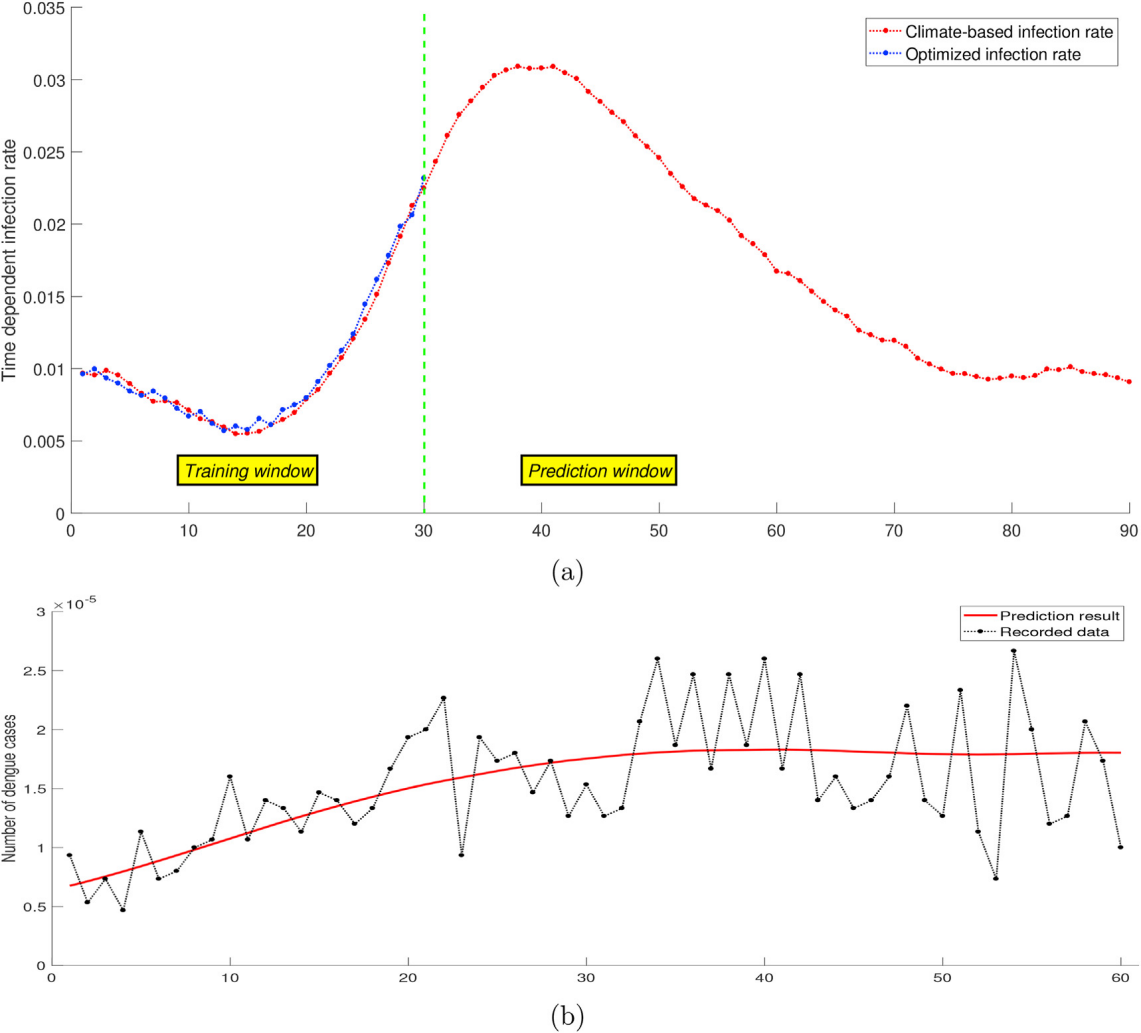
Forecasting dengue during *increasing period* (b) using extended infection rate (a).

**Fig. 7:**
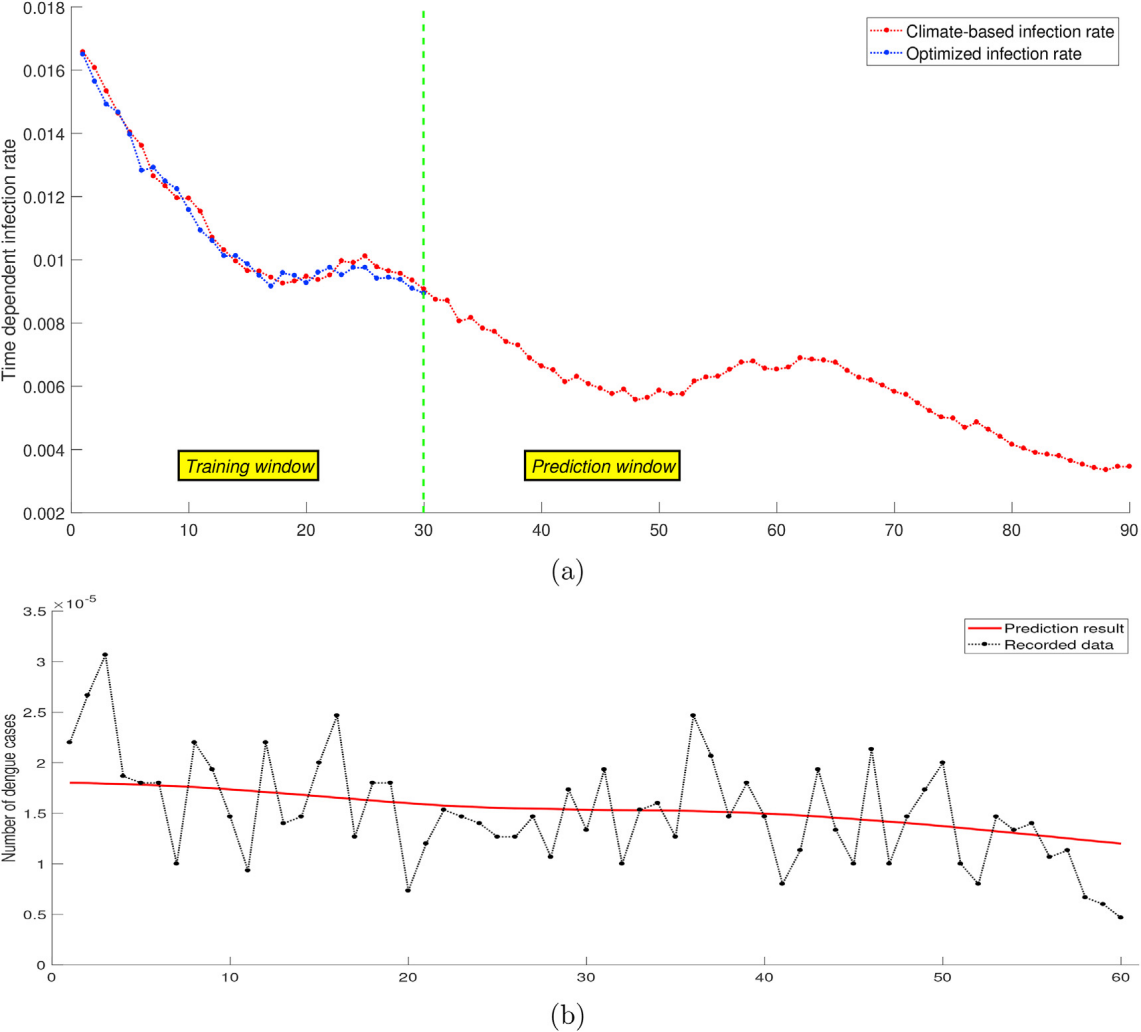
Forecasting dengue during *decreasing period* (b) using extended infection rate (a).

We examined further the performance of the ARDL model in making predictions of the infection rate parameter. If the updated daily relative humidity and precipitation data are known, then the infection rate *β*_*h*_ can be predicted using the model, and we can extend the observation time on the dengue transmission model to obtain the number of infected humans. Fig. 6 shows the prediction during dengue *increasing period*, and Fig. 7 shows the prediction during dengue *decreasing period*. Although the prediction result follows the dengue incidence data trend, it can perform inaccurate predictions for longer observation time because of the accuracy issue. For the short time interval, the biological model can catch at least the data pattern, and become a proper predictor of the dengue outbreak. A better understanding of identification of dengue outbreaks risk is a substantial basis to obtain early warning information, which is required by a public health agency to improve dengue preventions and interventions.

**Fig. 8:**
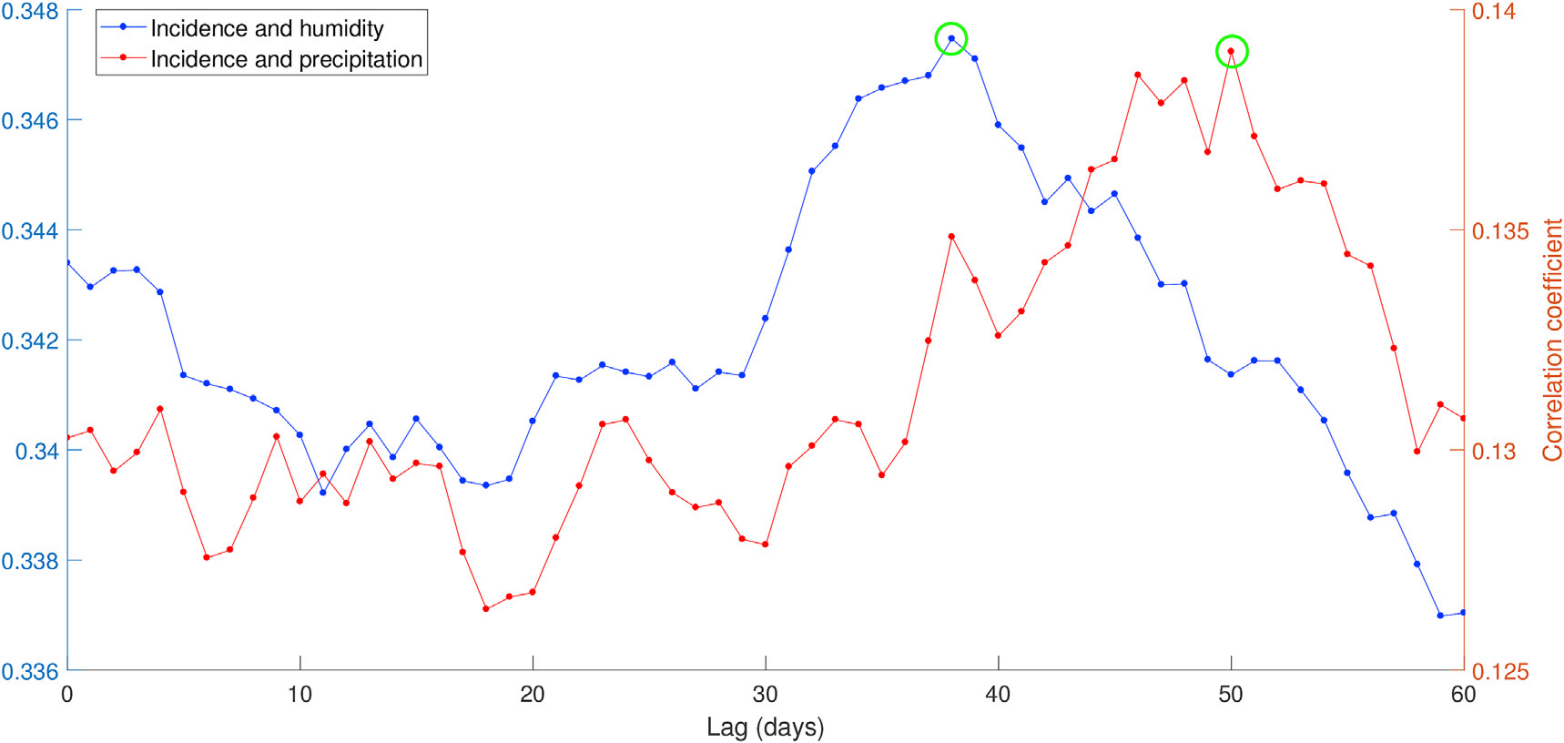
Cross correlation between dengue incidence, humidity, and precipitation.

Furthermore, we used descriptive analysis to describe the epidemiological characteristics of dengue incidences in Semarang during the observation time. The two-dimensional associations between dengue and meteorological variables were explored graphically, which explain dengue incidences mostly occurred in certain climatic conditions. Based on the cross-correlation of incidence-humidity and incidence-precipitation presented in Fig. 8, the lagged effects stand for 38 and 50 days, respectively. Moreover, the daily mean temperature is negatively correlated with dengue, and then we used lag zero in the exploration of the association between dengue incidence and temperature in Semarang.

Numerous researches identified the lagged effect of climatic factors on dengue fever incidence. Temperature, humidity, and precipitation can generate a cascade impact on the dengue outbreak by affecting the population size of *Aedes* mosquito. The lag between meteorological factors and incidence can be understood that it needs some days after the favorable weather to create an ideal habitat to support mosquito life cycle development and trigger the abundance of dengue transmission vector (Hii et al., 2016).

**Fig. 9:**
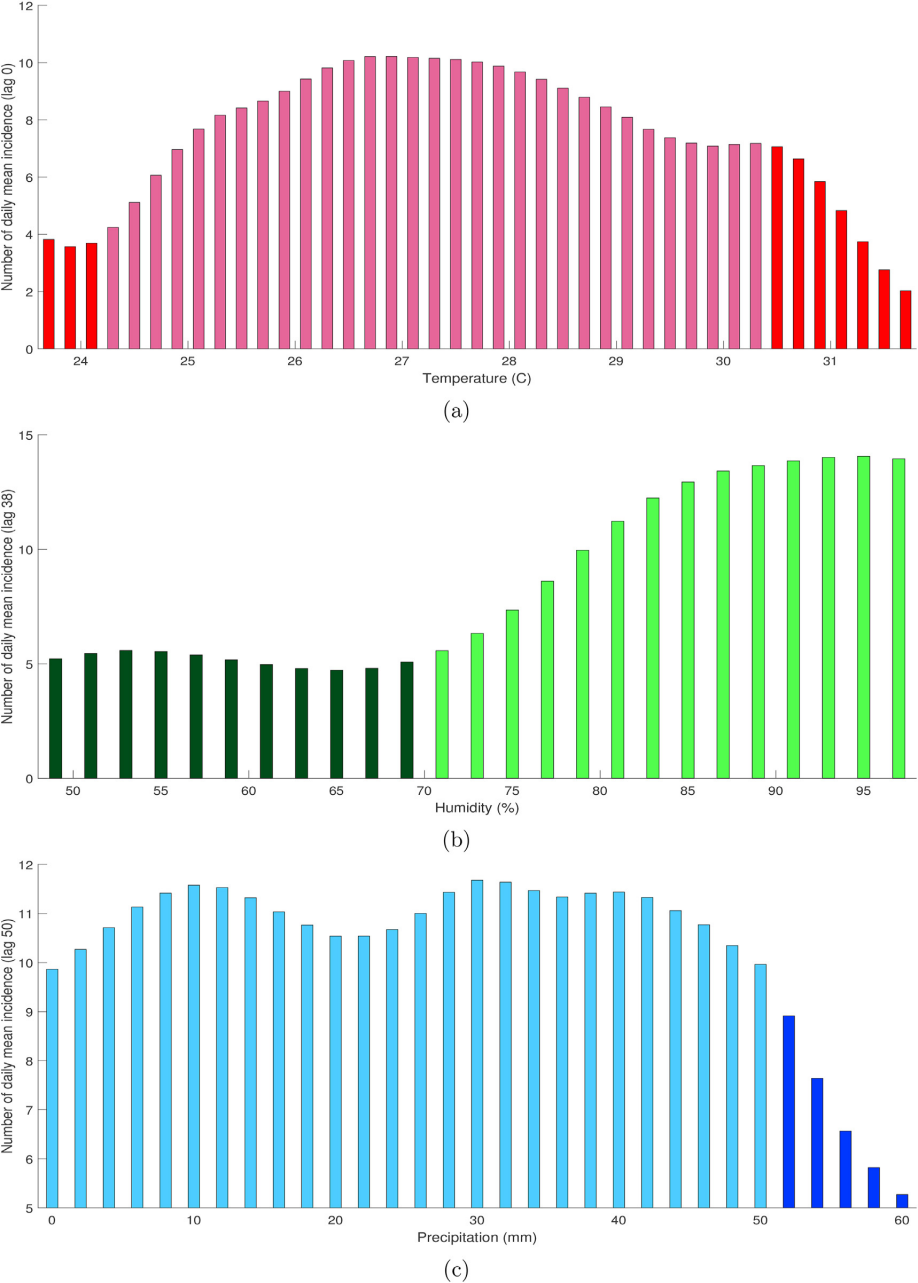
Association between daily mean incidence and temperature (a), humidity (b), and precipitation (c) at certain lag in Semarang with optimal range denoted by light color.

In Fig. 9a, the association between dengue incidence and temperature at lag 0 was graphically shown as a reversed U-shaped curve. The number of dengue cases increases with the increase of the temperature over 24.3 °C but decreases significantly when the temperature exceeded 30.5 °C. At lag 0, a 1 °C increase of temperature within the optimal range was associated with 9.67*%* increase in dengue incidence. Xiang et al. (Xiang et al., 2017) have identified similar threshold temperatures in Guangzhou, China with a range of maximum temperature 21.6–32.9 °C. Previously, Kakarla et al. (Kakarla et al., 2019) identified that positive correlation was observed between dengue and temperature for ranging between 24 °C and 30 °C at a lag of 0–4 weeks in India. Variations on the temperature within a certain range may shorten the maturation period from larva to adult mosquito (Lindsay & Mackenzie, 1997). It could also cause the acceleration of multiplication and maturation of the virus (Ebi & Nealon, 2016), and reduction of the extrinsic incubation period of dengue virus until it is transmissible to another human (Sharmin et al., 2015), and increase the frequency of female mosquito’s blood-feeding (Morin et al., 2013). The immature stages of mosquito development require minimal temperature 8.3–14 °C, the optimal temperature is about 30 °C, and immature development is inhibited when the temperature exceeds 36 °C (Colon-Gonzalez et al., 2013; Delatte et al., 2009; Morin et al., 2013). Adult *Aedes* mosquito can survive until air temperature reaches 40 °C (Christophers, 1960). The warmer temperature also may result in smaller adult mosquitoes that have high metabolism rates, which increase the frequency of blood-feeding and egg-laying (McMichael et al., 1996). Lastly, the temperature has on obvious influence on the length of the extrinsic incubation periods in which the duration of dengue viruses incubation inside mosquito body is 12 days at 30 °C and only 7 day at temperature condition 32–35 °C (Focks et al., 1995).

In Fig. 9b, a reversed slide-shaped curve association was obtained between dengue incidence and humidity. At lag 38, the daily percentage of relative humidity was positively associated with dengue upper the threshold 70*%*. A 1*%* increase of relative humidity was associated with a 4.06*%* increase in dengue on the optimal range. Throughout the year, the relative humidity in Semarang mostly remained at highly favorable levels (≥70*%*) with some fluctuations. By contrast, the negative association was observed during extremely humidity conditions, ≥ 78.9*%* in Guangzhou (Xiang et al., 2017). Inconsistent findings can be explained as the impact of diverse regional climate and environmental characteristic. The relative humidity is able to influence vector flight performance, oviposition and egg hatching (Xiang et al., 2017), mating pattern, and how male-female mosquitoes attract each other (Wu et al., 2007), dispersal, blood-feeding behavior (McMichael et al., 1996), and longevity of adult mosquito (Christophers, 1960; Ebi & Nealon, 2016).

Furthermore, in Fig. 9c, a reversed U-shaped association was also found between dengue incidence and precipitation. The number of dengue incidence generally increases with an increase of 24-h precipitation level below 50 mm. Rainfall expands breeding sites in nature, giving more space for the adult mosquito to lay the eggs (Lindsay & Mackenzie, 1997). The empty water containers provided by nature (e.g., bamboo tubes, rock pools, and tree holes) or human-made (e.g., uncovered barrels, water tanks, and beverage bottles) will be filled with the water when the rainfall occurs during the rainy season. The consequent expansion in the number of available *Aedes* breeding sites on nature favors the mosquito development in its egg, larva, and pupa stages, which occurs dominantly in the aquatic environment (Duarte et al., 2019; Viana & Ignotti, 2013). This process can potentially create the abundance of vector population and subsequently increase the risk of dengue infection. However, intense and heavy rainfall may erase the breeding place and reduce the mosquito population (Colon-Gonzalez et al., 2013; Putra & Nuraini, 2017). This study observed that the number of incidences declines when a 24-h precipitation level exceeds 50 mm. Besides, the availability of larva nurturing places in some areas is also directly influenced by domestic water storage habits. For example, the residents in low rainfall region usually store water in response to the possibility of drought period in which the number of mosquito breeding sites can elevate. Therefore, insufficient precipitation also can lead to increase vector densities and raise the risk of outbreak in the region (Jansen & Beebe, 2010).

## 4 Conclusions

Based on the given daily dengue data and the climate data in Semarang city, a SIR-SI model describing the host-vector dengue transmission dynamics was modified to model this situation. The climate factors were integrated into the model, in which the transmission parameters were depended on temperature, humidity, and precipitation. We considered the infection rate *β*_*h*_ as a time-dependent parameter to obtain well-approximated dengue incidence data. Spiral Dynamic Optimization was implemented to find the daily value of the infection parameter, which minimized the deviation between recorded data and simulation output. Furthermore, an ARDL model will be adapted to obtain a climate-based infection rate which associates climate factors and infection parameter. If the updated daily climatic factors are known, then infection rate *β*_*h*_ can be obtained for longer observation time, which is useful to forecast dengue outbreak in the future. However, the model does not give good predictions for the long term that may be influenced by the non-statistical significant value of the correlation between infection and climatic factors. Furthermore, the result of the descriptive analysis showed that a reversed slide-shaped curve association was observed for dengue-humidity and reversed U-shaped curve associated with the relationship between dengue-temperature and dengue-precipitation. Identified thresholds of climatic factors were required for early warning system and decision making concerning when to initiate prevention and control strategy.

This study has several limitations. The obtained dengue incidence data only performs the total number of hospitalized cases. However, the details of classification, e.g., gender, age structure, occupation, and address, are not available. The second limitation is the lack of variables differentiating indigenous and imported cases in the recorded data. Since we used the assumption for the biological model that the population is closed, imported dengue cases need to be excluded from data analysis. Thirdly, based on the obtained values of parameters in the ARDL model, the coefficients for humidity and precipitation are not significant that may be affected by the value of correlation (0.3670 for infection rate-humidity and 0.2056 for infection rate-precipitation). These facts indicate that the climatic factors sometimes do not follow the dengue data in determining whether to decrease or increase. Therefore, climatic variables alone can not stand as a good predictor for future dengue risk in long observation time. Finally, the diverse regional climates are able to exhibit a different relationship between dengue and climate factors, which may need different predictor variables in the ARDL model.

## Author contributions

**Nuning Nuraini**: Conceptualization, Formal Analysis, Investigation, Writing-Review and Editing. **Ilham Saiful Fauzi**: Conceptualization, Methodology, Software, Writing-Original Draft. **Muhammad Fakhruddin**: Methodology, Investigation, Visualization. **Ardhasena Sopaheluwakan**: Resources, Supervision. **Edy Soewono**: Investigation, Writing-Review and Editing, Supervision.

## Declaration of competing interest

The authors declare that they have no known competing financial interests or personal relationships that could have appeared to influence the work reported in this paper.
